# Test–Retest Reliability of Heart Rate and Parasympathetic Modulation Indices Across Exercise and Recovery Phases in Athletes

**DOI:** 10.3390/s26082448

**Published:** 2026-04-16

**Authors:** Süleyman Ulupınar, Serhat Özbay, Cebrail Gençoğlu, İzzet İnce, Salih Çabuk, Özgür Bakar, Abdullah Demirli, Kaan Kaya

**Affiliations:** 1 Faculty of Sports Sciences, Erzurum Technical University, Erzurum 25050, Türkiye; suleyman.ulupinar@erzurum.edu.tr (S.U.); serhat.ozbay@erzurum.edu.tr (S.Ö.); cebrail.gencoglu@erzurum.edu.tr (C.G.); salih.cabuk@erzurum.edu.tr (S.Ç.);; 2 Faculty of Sports Sciences, Ankara Yıldırım Beyazıt University, Ankara 06010, Türkiye; izzetince@aybu.edu.tr; 3 Faculty of Sports Sciences, Istanbul University-Cerrahpaşa, Istanbul 34320, Türkiye; abdullah.demirli@iuc.edu.tr; 4 Faculty of Sports Sciences, Istanbul Yeni Yuzyil University, Istanbul 34010, Türkiye

**Keywords:** heart rate variability, RMSSD, test–retest reliability, exercise recovery, wearable sensors, autonomic nervous system, parasympathetic modulation, soccer players

## Abstract

This study examined the within-session (same-day) test–retest reliability of heart rate (HR) and parasympathetic modulation, assessed using the root mean square of successive differences (RMSSD), across exercise and recovery phases in trained soccer players. Twenty-seven male soccer players (age: 24.9 ± 3.7 years) completed a standardized soccer training session. HR and RMSSD were recorded using an ECG-based chest-strap monitor at rest, pre-exercise, and at ~10–20 min, 1 h, and 3 h post-exercise. At each time point, two consecutive 5 min seated recordings were obtained under identical conditions. Test–retest reliability was evaluated using intraclass correlation coefficients (ICC_(3,1)_), standard error of measurement (SEM), coefficient of variation (CV%), minimal detectable change (MDC_95_), paired-samples *t*-tests, and Hedges’ g effect sizes. HR demonstrated excellent reliability across all time points (ICC = 0.980–0.994; SEM = 0.87–1.25 bpm; CV% = 1.33–3.70%). RMSSD showed excellent reliability at rest (ICC = 0.944) and pre-exercise (ICC = 0.918), moderate reliability during early recovery (~10–20 min; ICC = 0.551), and good reliability at 1 h (ICC = 0.826) and 3 h post-exercise (ICC = 0.873). No significant systematic differences were observed between test and retest measurements (all *p* > 0.05), and effect sizes were trivial. These findings indicate that within-session reliability of HR remains consistently high across exercise and recovery phases, whereas RMSSD reliability varies according to measurement timing, particularly during early recovery.

## 1. Introduction

Heart rate variability (HRV) has become a widely used non-invasive marker for assessing autonomic nervous system regulation in both clinical and sports science settings [1,2,3]. In particular, HRV-derived indices provide valuable insight into the balance between sympathetic and parasympathetic modulation, which is closely linked to cardiovascular control, fatigue, and recovery processes [4,5]. Among time-domain HRV parameters, the root mean square of successive differences (RMSSD) is considered a robust surrogate of parasympathetic activity due to its sensitivity to vagal modulation and relative resistance to respiratory influences [6,7,8]. Owing to these characteristics, RMSSD has been extensively adopted in athletic populations for monitoring training load, recovery status, and physiological adaptation, especially in field-based and applied sport environments [9,10,11].

Recent advances in wearable sensor technology have substantially increased the feasibility of continuous heart rate (HR) and HRV monitoring in applied sport settings [2,3,12]. Chest-strap monitors and ECG-derived wearable devices are now widely used due to their practicality, portability, and ability to provide real-time physiological feedback during training and recovery [13,14,15]. However, beyond device validity, the reliability of repeated measurements remains a critical methodological requirement for meaningful data interpretation. Test–retest reliability is particularly important when HRV indices are used to track short-term physiological fluctuations or to inform day-to-day training decisions [15,16,17,18]. Variability arising from measurement noise, signal processing, or transient physiological perturbations may compromise the interpretability of HRV-derived metrics, underscoring the need to establish reliable measurement properties under standardized conditions [16,19].

Despite the widespread use of HRV monitoring, most reliability studies have primarily examined measurements obtained under resting conditions, where autonomic regulation is relatively stable [1,9,16]. However, HRV indices—particularly vagally mediated parameters such as RMSSD—have also been employed to characterize autonomic recovery following acute exercise [9,12,20]. Previous investigations have demonstrated that post-exercise HRV reflects the kinetics of parasympathetic reactivation and sympathetic withdrawal, thereby providing insight into recovery dynamics after physical exertion [21,22]. Nevertheless, although HRV has been used to describe post-exercise autonomic responses, the test–retest reliability of these measures during the recovery phase has not been systematically established. The recovery period is characterized by rapid and non-linear shifts in autonomic balance, which may influence the stability of HRV-derived parameters such as RMSSD [23,24,25]. These transient physiological fluctuations may increase within-subject variability and potentially reduce measurement consistency, particularly during the early phases of recovery [5,26,27]. Consequently, the extent to which RMSSD can be reliably assessed across different post-exercise time points remains insufficiently understood, especially when compared with the typically more stable behavior of heart rate measurements.

Therefore, the purpose of the present study was to examine the test–retest reliability of heart rate and parasympathetic modulation, assessed via RMSSD, across distinct exercise and recovery phases in trained soccer players. Heart rate and RMSSD were evaluated at rest, prior to exercise, and at multiple post-exercise recovery time points to capture phase-specific reliability profiles. By directly comparing the reliability characteristics of HR and RMSSD across these conditions, this study aims to clarify whether parasympathetic indices derived from wearable, ECG-based sensors can be interpreted consistently throughout the recovery process. The findings are expected to provide practical guidance for researchers and practitioners regarding the appropriate use of RMSSD in post-exercise monitoring and contribute to a more nuanced understanding of HRV-based recovery assessment in athletic populations.

## 2. Methods

### 2.1. Participants

Twenty-seven male trained soccer players volunteered to participate in this study (age: 24.9 ± 3.74 years; height: 176.3 ± 5.15 cm; body mass: 73.1 ± 4.78 kg). All participants were actively competing and regularly training, with a routine schedule consisting of structured team training sessions and one official match per week.

To evaluate the adequacy of the sample size for reliability analysis, a post hoc power analysis was conducted using G*Power (v3.1.9.4) based on correlation statistics (bivariate normal model, two-tailed). As a conservative approach, the lowest observed reliability coefficient in the study (ICC = 0.551, obtained for RMSSD during early post-exercise recovery) was entered as the expected effect size (ρ = 0.551), with α = 0.05 and total sample size *n* = 27. The achieved statistical power was 0.87 (1 – β = 0.872), indicating sufficient sensitivity to detect moderate reliability coefficients within the present sample. Although the interpretative value of post hoc power analysis is debated in methodological literature, it was included here as a supplementary indicator of sample adequacy. Importantly, intraclass correlation coefficients and their confidence intervals remain the primary metrics for evaluating measurement precision in reliability research [28,29,30,31].

Inclusion criteria were: (i) being an active soccer player with regular training participation, (ii) free from musculoskeletal injury that could limit training participation within the previous months, and (iii) absence of known cardiovascular, metabolic, or neurological disease and no use of medications or substances known to affect autonomic function. Prior to participation, all athletes were informed about the procedures and provided written informed consent.

The study was approved by the Erzurum Technical University Scientific Research and Publication Ethics Committee (Meeting No: 06; Decision No: 18; Date: 21 April 2025) and conducted in accordance with the principles of the Declaration of Helsinki.

### 2.2. Study Design

**Figure 1:**
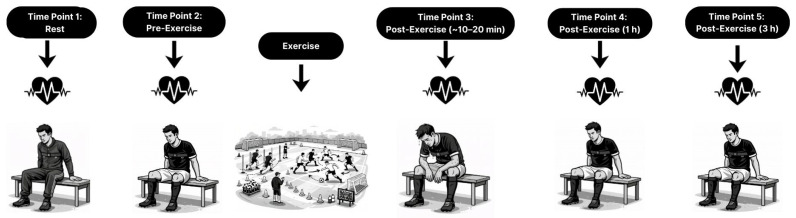
Study design illustrating measurement time points and test–retest recording structure. At each predefined time point, two consecutive 5 min seated RR recordings were obtained, separated by a standardized 1 min interval.

This study employed a within-subject test–retest reliability design to examine the consistency of heart rate and HRV-derived parasympathetic indices across distinct exercise and recovery phases (Figure 1). All measurements were performed on the same day under strictly standardized conditions to minimize biological variability unrelated to the measurement process. Test and retest recordings were obtained consecutively at each time point and were separated by a fixed 1 min interval, during which participants maintained an identical body posture and environmental setting. Data collection was conducted following a typical soccer training session designed to induce substantial cardiovascular and autonomic perturbations. To control for residual fatigue and competition-related stress, all testing sessions were scheduled 72–96 h after the most recent official match. Participants were instructed to refrain from vigorous physical activity, alcohol, and stimulant consumption for at least 24 h prior to testing and to maintain their habitual sleep, hydration, and dietary routines throughout the testing period.

Heart rate and HRV measurements were obtained at five predefined time points: rest, pre-exercise, and ~10–15 min, 1 h, and 3 h post-exercise. At each time point, two consecutive recordings were performed for test–retest analysis. All measurements were conducted in a seated position under standardized conditions. Resting and pre-exercise recordings were obtained following a brief stabilization period, while post-exercise measurements were collected during passive recovery with participants remaining seated, ensuring consistent posture and minimizing postural influences on autonomic regulation. All measurements were performed in a quiet indoor environment with stable ambient conditions.

### 2.3. Exercise Protocol

Participants completed a standardized soccer training session designed to induce a representative cardiovascular and autonomic load typical of competitive practice. The session included a structured warm-up, followed by small-sided games (SSGs), intermittent high-intensity running and sprint drills, and sport-specific technical exercises involving passing, positional play, and directional changes. This combination was selected to elicit repeated fluctuations in heart rate and autonomic activity, reflecting the intermittent and multidirectional nature of soccer performance.

The training session lasted approximately 70–90 min, consistent with routine in-season soccer training, and was conducted under the supervision of the team coaching staff. No experimental pacing or workload manipulation was imposed; instead, players performed all drills at their habitual training intensity to preserve ecological validity.

Training load was quantified using mean heart rate (HR), peak HR, and session rating of perceived exertion (sRPE; Borg CR20 scale). The mean HR during the session was 149.0 ± 6.1 bpm, with peak values reaching 179.1 ± 5.8 bpm. The mean sRPE score was 16 ± 1.6 arbitrary units, indicating a moderate-to-high internal training load. These values confirm that the session elicited substantial cardiovascular stress while remaining within the range of typical competitive training demands.

Immediately following the completion of the training session, participants transitioned to a passive recovery period during which post-exercise measurements were obtained. No active recovery strategies, stretching routines, or external recovery modalities were permitted. All post-exercise assessments were conducted under standardized seated conditions, ensuring that observed changes in heart rate and HRV reflected physiological recovery dynamics rather than movement- or intervention-related influences.

### 2.4. Heart Rate and HRV Measurements

Heart rate (HR) and heart rate variability (HRV) data were recorded using a Polar H10 chest-strap heart rate monitor (Polar Electro Oy, Kempele, Finland), an ECG-based wearable device validated for accurate RR interval detection under both resting and exercise conditions. The Polar H10 records ECG signals at a sampling frequency of 1000 Hz and provides RR interval data with 1 ms resolution. Previous investigations have demonstrated high agreement between the Polar H10 and standard electrocardiography systems for short-term HRV assessment in athletic populations [14,32]. The device was positioned according to the manufacturer’s guidelines to ensure stable skin contact and optimal signal quality throughout all recordings. Prior to each measurement, signal integrity was visually inspected to minimize artifacts related to electrode displacement or movement.

All HR and HRV recordings were obtained under standardized, seated conditions. For each measurement, RR intervals were recorded continuously over a 5 min seated recording period, which is considered the standard duration for short-term HRV analysis [8,19]. Participants were instructed to remain still, breathe spontaneously, and avoid speaking or unnecessary movement during data acquisition.

Raw RR interval data were exported and processed using Kubios HRV software (version 3.5, University of Eastern Finland, Kuopio, Finland), a validated platform for standardized HRV analysis [33]. Artifact correction was performed using the automatic correction algorithm with the medium filter setting. Following automatic filtering, all recordings were visually inspected to confirm appropriate identification of normal-to-normal (NN) intervals. Recordings with excessive artifact correction (>5% corrected beats) were excluded from analysis. Only NN intervals were retained for subsequent HRV calculations.

RMSSD was selected due to its established sensitivity to parasympathetic modulation and suitability for short-duration recordings in applied settings [10,34]. Heart rate was calculated as the mean beats per minute over the same 5 min recording period for each measurement. Both HR and RMSSD values were derived identically for test and retest recordings at each predefined time point, ensuring methodological consistency across all exercise and recovery phases.

### 2.5. Statistical Analysis

All statistical analyses were performed using the Simplified Statistical Program (KIP, version 1.0), a statistical software developed to facilitate standardized analysis and reporting in academic research [35]. To ensure transparency and reproducibility, all primary statistical analyses, including intraclass correlation coefficients, paired-samples *t*-tests, and effect size calculations, were independently replicated using SPSS (version 27.0). Identical test statistics, *p*-values, and effect size estimates were obtained across platforms.

Prior to inferential analyses, the assumption of normality was evaluated using the Shapiro–Wilk test and visual inspection of Q–Q plots for both HR and RMSSD at each measurement time point. No significant deviations from normality were detected (all *p* > 0.05); therefore, parametric statistical procedures were deemed appropriate.

Test–retest reliability was assessed using intraclass correlation coefficients (ICC) calculated with a two-way mixed-effects model, single measurement, and absolute agreement definition (ICC_(3,1)_). This model was selected because the same device and standardized measurement protocol were applied across all sessions, and the primary objective was to evaluate absolute agreement between single 5 min test and retest recordings at each time point rather than consistency or averaged measurements [29,36]. ICC values were interpreted as poor (<0.50), moderate (0.50–0.75), good (0.75–0.90), or excellent (>0.90).

To further quantify absolute reliability, the standard error of measurement (SEM) was calculated using the ICC-based approach as SEM = SD_pooled × √(1 − ICC), where SD_pooled was computed as √[(SD_test^2^ + SD_retest^2^)/2]. The minimal detectable change at the 95% confidence level (MDC_95_) was calculated as MDC_95_ = SEM × 1.96 × √2. The coefficient of variation (CV%) was computed as (SEM/grand mean) × 100, where the grand mean was defined as the average of test and retest means at each time point [29,36]. This SEM-based CV% reflects absolute reliability (measurement error relative to the mean) rather than dispersion of raw scores (i.e., SD/mean × 100), thereby aligning with reliability-focused reporting frameworks.

Systematic differences between test and retest values were evaluated using paired-samples *t*-tests, and the magnitude of these differences was quantified using Hedges’ g effect sizes. Effect sizes were interpreted according to the scale proposed by Hopkins as trivial (<0.20), small (0.20–0.59), moderate (0.60–1.19), large (1.20–1.99), very large (2.00–3.99), and nearly perfect (≥4.00) [28,30]. The Hopkins classification was selected for its widespread use in sports science research, particularly in performance and monitoring contexts, where sensitivity to small yet practically meaningful effects is emphasized [28,29,30]. Statistical significance was set at *p* < 0.05 for all analyses. Descriptive statistics are presented as mean ± standard deviation (SD).

## 3. Results

Heart rate test–retest reliability across measurement time points is presented in Table 1. Intraclass correlation coefficients ranged from 0.980 to 0.994. Reliability was classified as excellent at rest (ICC = 0.980, 95% CI: 0.956–0.991) and pre-exercise (ICC = 0.980, 95% CI: 0.956–0.991). During early post-exercise recovery (~10–20 min), the ICC was 0.984 (95% CI: 0.965–0.993). At 1 h and 3 h post-exercise, ICC values were 0.994 (95% CI: 0.987–0.997) at both time points. SEM values ranged from 0.87 to 1.25 bpm across measurement conditions. CV% values ranged from 1.33% to 3.70%. MDC_95_ values ranged from 2.41 to 3.47 bpm.

Paired-samples *t*-test analyses revealed no significant systematic differences between test and retest heart rate measurements at any of the assessed time points (*p* > 0.05 for all comparisons, Table 2). Mean heart rate values were highly comparable between test and retest conditions across rest, pre-exercise, and post-exercise measurements. The magnitude of test–retest differences was consistently trivial, with Hedges’ g values ranging from −0.08 to 0.19. At rest, the difference between test (57.2 ± 6.3 bpm) and retest (57.1 ± 6.5 bpm) measurements was negligible (t = 0.440, *p* = 0.663, g = −0.08). Similarly, no meaningful differences were observed during pre-exercise (g = 0.17), early post-exercise recovery (~10–20 min; g = 0.19), or later recovery phases at 1 h (g = 0.17) and 3 h (g = −0.03).

RMSSD test–retest reliability across measurement time points is presented in Table 3. At rest, reliability was excellent (ICC = 0.944, 95% CI: 0.880–0.974, *p* < 0.001). Pre-exercise reliability was also excellent (ICC = 0.918, 95% CI: 0.829–0.962, *p* < 0.001). During early post-exercise recovery (~10–20 min), reliability decreased to a moderate level (ICC = 0.551, 95% CI: 0.228–0.766, *p* < 0.001). At 1 h post-exercise, reliability was classified as good (ICC = 0.826, 95% CI: 0.654–0.917, *p* < 0.001), and at 3 h post-exercise, reliability was good (ICC = 0.873, 95% CI: 0.737–0.941, *p* < 0.001). The SEM values ranged from 2.22 to 4.32 ms across time points. The CV% values ranged from 3.79% to 4.54%. The MDC_95_ values ranged from 6.16 to 11.97 ms.

Paired-samples *t*-test analyses revealed no significant systematic differences between test and retest RMSSD values at any measurement time point (*p* > 0.05 for all comparisons, Table 4). Mean RMSSD values were highly comparable between repeated measurements across rest, pre-exercise, and post-exercise recovery phases. The magnitude of test–retest differences was consistently trivial, with Hedges’ g values ranging from 0.01 to 0.18 according to Hopkins’ effect size classification. At rest, the difference between test (68.15 ± 13.52 ms) and retest (68.19 ± 12.58 ms) measurements was negligible (t = −0.043, *p* = 0.966, g = 0.01). Similarly, trivial effect sizes were observed during pre-exercise (g = 0.05), early post-exercise recovery (~10–20 min; g = 0.18), and later recovery phases at 1 h (g = 0.10) and 3 h (g = 0.01).

## 4. Discussion

The primary finding of the present study is that heart rate and parasympathetic modulation indices exhibit fundamentally different test–retest reliability profiles across exercise and recovery phases. While heart rate demonstrated consistently excellent reliability at all measurement time points—ranging from rest to early and late post-exercise recovery—RMSSD showed a clear phase-dependent pattern. Specifically, RMSSD exhibited excellent reliability under resting and pre-exercise conditions, moderate reliability during early recovery (~10–20 min post-exercise), and good reliability at later recovery time points (1 h and 3 h post-exercise). This divergence highlights the inherent stability of heart rate as a cardiovascular marker, in contrast to the greater physiological sensitivity of RMSSD, which appears to be influenced by transient autonomic fluctuations occurring during recovery. Importantly, these findings underscore that the reliability of HRV-derived parasympathetic indices cannot be assumed to be uniform across physiological states, even when measurements are obtained under standardized conditions using ECG-based wearable devices.

The reduced test–retest reliability of RMSSD observed during early recovery (~10–20 min post-exercise) is most plausibly explained by the rapid and heterogeneous autonomic adjustments that occur immediately following exercise cessation. RMSSD is widely recognized as a sensitive index of parasympathetic modulation, and its values are known to fluctuate markedly during periods of acute physiological transition [8,37]. Previous research has consistently shown that acute exercise induces substantial vagal withdrawal, followed by a non-linear and highly individualized parasympathetic reactivation during early recovery [26]. As a consequence, even under standardized postural and environmental conditions, short-term HRV measures may exhibit increased within-subject variability during this phase.

Importantly, several studies have demonstrated that HRV reliability is strongly state-dependent, with lower reproducibility observed during transient or unstable physiological conditions. For example, moderate reliability has been reported for short-term HRV recordings during acute recovery or altered physiological states, despite acceptable reliability under resting conditions [16,38]. Similar state-dependent effects have also been documented in sleep research, where HRV reliability varies across sleep stages and during disrupted sleep, further supporting the notion that autonomic instability compromises measurement consistency rather than signal quality [17]. In contrast, studies conducted under stable resting conditions using standardized 5 min ECG recordings consistently report excellent RMSSD reliability (ICC ≈ 0.90–0.95), reinforcing that reduced reliability during early recovery is unlikely to reflect methodological shortcomings [19].

Collectively, these findings suggest that the moderate RMSSD reliability observed during early recovery may be related to increased physiological variability associated with rapid autonomic adjustments following exercise. However, measurement noise and uncontrolled respiratory influences cannot be excluded as potential contributing factors. Previous literature indicates that HRV metrics, particularly RMSSD, are sensitive to acute psychophysiological perturbations [25,39], and meta-analytic evidence has shown that HRV reliability may vary depending on recording context and physiological state, even when methodological factors such as recording duration and signal acquisition are standardized [18].

An additional methodological consideration relates to the absence of respiratory monitoring during HRV recordings. Although participants were instructed to breathe spontaneously under standardized seated conditions, respiratory frequency was not directly measured or controlled. It is well established that RMSSD, while relatively robust among time-domain HRV indices, remains influenced by respiratory patterns due to respiratory sinus arrhythmia [1,16,22]. Variations in breathing rate and tidal volume may alter short-term vagally mediated HRV indices, particularly under conditions of heightened physiological instability [11,22]. Early post-exercise recovery is characterized not only by rapid autonomic reorganization but also by elevated and progressively normalizing ventilatory responses. Consequently, inter- and intra-individual variability in spontaneous breathing patterns during this phase may have contributed to the moderate ICC values observed at ~10–20 min post-exercise. Importantly, the lack of systematic bias between test and retest measurements suggests that respiratory variability likely increased within-subject dispersion rather than introducing directional measurement error. Nevertheless, the absence of respiratory control should be acknowledged as a potential confounding factor when interpreting phase-specific RMSSD reliability during early recovery.

The absence of significant systematic differences between test and retest measurements further supports the interpretation that the observed reductions in RMSSD reliability during early recovery are not attributable to measurement error or methodological bias. Across all time points, paired-samples analyses revealed non-significant mean differences with trivial effect sizes according to Hopkins’ classification, indicating a lack of consistent over- or underestimation between repeated recordings. This finding is critical, as reduced relative reliability (i.e., lower ICC values) in the absence of systematic bias typically reflects increased within-subject biological variability rather than poor measurement precision. Similar dissociations between relative reliability indices and absolute agreement have been reported in previous HRV studies, particularly under conditions characterized by transient autonomic instability [16,38].

From a methodological perspective, the use of a two-way mixed-effects ICC model with absolute agreement, combined with standardized seated posture, controlled environmental conditions, and 5 min ECG-based recordings, provides a robust framework for evaluating true test–retest reliability. The present results are consistent with prior studies reporting excellent RMSSD reproducibility under stable resting conditions when comparable methodological rigor is applied [8,19]. The moderate ICC values observed during early recovery should therefore be interpreted in the context of the rapidly changing autonomic state characteristic of this phase. Similarly, the good reliability observed at 1 h and 3 h post-exercise indicates partial stabilization of autonomic modulation compared with the early recovery phase. However, potential contributions of measurement noise and uncontrolled respiratory variability cannot be fully excluded. This distinction is important for both researchers and practitioners, as it highlights that reliability metrics must be interpreted within the physiological context in which measurements are obtained rather than considered in isolation.

From a technological perspective, the present findings provide further support for the use of ECG-based wearable systems in the assessment of heart rate and short-term HRV in applied sport settings. The consistently excellent reliability observed for heart rate across all phases, together with the high RMSSD reliability under resting and later recovery conditions, indicates that the wearable ECG platform used in this study is capable of delivering stable and reproducible measurements when physiological conditions are relatively steady. These results align with previous research demonstrating that ECG-based wearables can achieve reliability levels comparable to laboratory-grade systems, particularly for time-domain HRV indices derived from standardized 5 min recordings [8,19].

The phase-dependent variability observed for RMSSD should be interpreted within the context of the measurement system and physiological state. In the present study, HR and RR intervals were recorded using the Polar H10, an ECG-based chest-strap device that has demonstrated high agreement with standard electrocardiography for short-term RR interval detection during both rest and exercise conditions [14,32]. ECG-based wearable systems provide direct detection of cardiac electrical activity, which supports accurate beat-to-beat interval acquisition. The observed differences in reliability across recovery phases coincide with periods characterized by rapid shifts in autonomic balance. Previous literature has shown that wearable-derived HRV metrics are sensitive to acute psychophysiological perturbations, resulting in measurable variability under dynamic physiological conditions [25,39]. The present findings therefore indicate that reliability estimates may vary depending on the physiological phase being assessed. This consideration is relevant for sensor-based monitoring applications, particularly when HRV is used to evaluate recovery status across distinct autonomic states.

The present findings also have implications for wearable sensor selection in applied settings. The Polar H10 is an ECG-based chest-strap device that detects cardiac electrical activity directly, providing accurate beat-to-beat interval acquisition [14,32]. In contrast, photoplethysmography (PPG)-based wearables estimate heart rate and HRV indirectly through peripheral blood volume changes, which are more susceptible to motion artifacts, peripheral vasoconstriction, and signal distortion, particularly during and immediately after exercise [3,6]. Therefore, reliability estimates observed in the present study using an ECG-based device may not directly translate to PPG-based systems. These distinctions are relevant within the broader context of wearable sensor development and validation, particularly for applications involving dynamic recovery monitoring.

From a practical standpoint, the present findings carry important implications for the use of heart rate and HRV metrics in daily training monitoring and recovery assessment. The consistently high (excellent) reliability observed for heart rate across all measurement phases indicates that HR can be reliably applied as a stable indicator of cardiovascular load and recovery status within the same session. In contrast, the phase-dependent reliability of RMSSD suggests that practitioners should exercise caution when interpreting short-term HRV values obtained during early post-exercise recovery (~10–20 min). During this period, reduced reliability and greater within-subject variability may occur, potentially leading to over- or underestimation of autonomic recovery status if RMSSD is used in isolation. For applied settings such as athlete readiness assessment, load management, and return-to-training decisions, these findings highlight the importance of measurement timing. RMSSD appears to provide more reliable information when assessed under stable physiological conditions, such as at rest and pre-exercise (where excellent reliability was observed) and during later recovery phases (where reliability was classified as good), whereas heart rate remains reliable even during acute recovery. Consequently, combining heart rate with HRV-derived indices and standardizing the timing of measurements may enhance decision-making accuracy and reduce the risk of false-positive or false-negative interpretations. This approach is particularly relevant for wearable-based monitoring systems, where frequent data collection is feasible but requires informed contextualization to distinguish meaningful physiological signals from transient autonomic fluctuations.

### Limitations and Future Directions

Several limitations of the present study should be acknowledged. First, the sample consisted exclusively of male, regularly trained soccer players, which may limit the generalizability of the findings to female athletes, untrained individuals, or populations from other sport disciplines. Second, test–retest reliability was assessed within the same day using consecutive recordings separated by a short interval. Although appropriate for evaluating short-term measurement stability across physiological phases, this design reflects within-session technical reliability under controlled conditions and does not allow conclusions regarding day-to-day reproducibility or long-term reliability across separate training sessions. Therefore, the findings should not be directly generalized to longitudinal athlete monitoring applications. Third, although measurements were obtained under standardized seated conditions, respiratory frequency was not directly controlled or recorded. While RMSSD is generally considered less sensitive to variations in breathing rate compared to frequency-domain HRV indices, respiratory patterns can still influence short-term vagally mediated HRV measures. Therefore, uncontrolled breathing may have contributed to additional variability across recovery phases, particularly during periods of rapid autonomic adjustment.

Future research should aim to extend these findings by examining phase-dependent reliability of HRV metrics across different sports, training statuses, and sex groups. Investigations incorporating controlled breathing protocols, alternative postures, or varying recording durations may further clarify the interaction between physiological state and HRV reliability. Additionally, longitudinal designs evaluating day-to-day and week-to-week reliability following exercise would provide valuable insights for applied monitoring frameworks. Such efforts will help refine best-practice guidelines for interpreting wearable-derived HRV metrics and enhance their utility in both research and performance-oriented environments.

## 5. Conclusions

This study examined within-session (same-day) test–retest reliability of heart rate and RMSSD across rest, exercise, and recovery phases using an ECG-based wearable system. Heart rate demonstrated excellent reliability at all measurement time points. In contrast, RMSSD showed phase-dependent reliability, with excellent values at rest and pre-exercise, moderate reliability during early recovery (~10–20 min post-exercise), and good reliability during later recovery phases (1 h and 3 h post-exercise).

No systematic differences were observed between repeated measurements. These findings indicate that reliability estimates for HR and RMSSD vary across measurement phases within the same session. Accordingly, the present results support the use of ECG-based wearable technology for within-session assessment of heart rate and short-term HRV, while highlighting that RMSSD reliability may differ depending on the timing of measurement during recovery.

## Figures and Tables

**Table 1:** Test–Retest Reliability of Heart Rate: Intraclass Correlation Coefficients and Absolute Reliability Indices.

Time Point	ICC_(3,1)_ (95% CI)	*p*-Value	Interpretation	SEM	CV%	MDC_95_
Rest	0.980 (0.956–0.991)	<0.001	Excellent	0.91	1.33	2.51
Pre-Exercise	0.980 (0.956–0.991)	<0.001	Excellent	0.87	1.43	2.41
Post-Exercise (~10–20 min)	0.984 (0.965–0.993)	<0.001	Excellent	1.25	3.70	3.47
Post-Exercise (1 h)	0.994 (0.987–0.997)	<0.001	Excellent	0.92	2.08	2.56
Post-Exercise (3 h)	0.994 (0.987–0.997)	<0.001	Excellent	0.95	1.57	2.62

*Note*. ICC_(3,1)_ = intraclass correlation coefficient calculated using a two-way mixed-effects model, single measurement, absolute agreement definition; 95% CI = 95% confidence interval; *p*-value = statistical significance level associated with the ICC; SEM = standard error of measurement; CV% = coefficient of variation; MDC_95_ = minimal detectable change at the 95% confidence level.

**Table 2:** Paired-Samples *t*-Test and Hedges’ g Effect Sizes for Test–Retest Differences in Heart Rate.

Time Point	Test	Retest	t-Value	*p*-Value	Hedges’ g
Rest	57.2 ± 6.3	57.1 ± 6.5	0.440	0.663	−0.08, trivial
Pre-Exercise	68.9 ± 6.0	69.1 ± 6.3	−0.923	0.364	0.17, trivial
Post-Exercise (~10–20 min)	100.9 ± 9.9	101.3 ± 9.9	−1.082	0.289	0.19, trivial
Post-Exercise (1 h)	82.7 ± 12.0	82.9 ± 11.8	−0.881	0.386	0.17, trivial
Post-Exercise (3 h)	69.2 ± 12.2	69.2 ± 12.2	0.143	0.887	−0.03, trivial

**Table 3:** Test–Retest Reliability of RMSSD: Intraclass Correlation Coefficients and Absolute Reliability Indices.

Time Point	ICC_(3,1)_ (95% CI)	*p*-Value	Interpretation	SEM	CV%	MDC_95_
Rest	0.944 (0.880–0.974)	<0.001	Excellent	2.22	3.89	6.16
Pre-Exercise	0.918 (0.829–0.962)	<0.001	Excellent	3.13	4.54	8.67
Post-Exercise (~10–20 min)	0.551 (0.228–0.766)	<0.001	Moderate	4.32	4.27	11.97
Post-Exercise (1 h)	0.826 (0.654–0.917)	<0.001	Good	3.42	4.13	9.48
Post-Exercise (3 h)	0.873 (0.737–0.941)	<0.001	Good	2.63	3.79	7.28

*Note*. ICC_(3,1)_ = intraclass correlation coefficient calculated using a two-way mixed-effects model, single measurement, absolute agreement definition; 95% CI = 95% confidence interval; *p*-value = statistical significance level associated with the ICC; SEM = standard error of measurement; CV% = coefficient of variation; MDC_95_ = minimal detectable change at the 95% confidence level.

**Table 4:** Paired-Samples *t*-Test and Hedges’ g Effect Sizes for Test–Retest Differences in RMSSD.

Time Point	Test	Retest	t-Value	*p*-Value	Hedges’ g
Rest	68.2 ± 13.5	68.2 ± 12.6	−0.043	0.966	0.01, trivial
Pre-Exercise	60.7 ± 13.3	61.0 ± 16.4	−0.283	0.779	0.05, trivial
Post-Exercise (~10–20 min)	33.2 ± 6.2	34.6 ± 9.5	−0.986	0.333	0.18, trivial
Post-Exercise (1 h)	44.0 ± 9.4	44.6 ± 12.3	−0.531	0.600	0.10, trivial
Post-Exercise (3 h)	60.2 ± 9.8	60.2 ± 10.4	−0.038	0.970	0.01, trivial

## Data Availability

The data presented in this study are available from the corresponding author upon reasonable request.
